# The Impact of Biseasonal Time Changes on Migraine

**DOI:** 10.3390/neurolint17030040

**Published:** 2025-03-05

**Authors:** Carl H. Göbel, Katja Heinze-Kuhn, Axel Heinze, Anna Cirkel, Hartmut Göbel

**Affiliations:** 1 Kiel Migraine and Headache Center, 24149 Kiel, Germany; khk@schmerzklinik.de (K.H.-K.); heinze@schmerzklinik.de (A.H.); anna.cirkel@uni-luebeck.de (A.C.); hg@schmerzklinik.de (H.G.); 2 Department of Neurology, University Hospital Schleswig-Holstein, Campus Kiel, 24149 Kiel, Germany; 3 Department of Neurology, University Hospital Schleswig-Holstein, Campus Lübeck, 23562 Lübeck, Germany

**Keywords:** biseasonal time change, chronobiology, circadian rhythmic, standard time, daylight savings time, migraine, headache

## Abstract

**Background**: Changes in the daily rhythm can trigger migraine attacks. The sensitivity for triggering attacks is closely linked to the regulation of biological rhythms controlled by the hypothalamus. In over 70 countries around the world, the time is changed between daylight savings time and standard time twice a year due to legal regulations. The aim of this study was to investigate whether the time change has an influence on migraine. **Methods**: In this retrospective study, the headache frequency of patients with episodic or chronic migraine at a tertiary headache center in the years 2020, 2021, and 2022 was evaluated. The primary outcome measure was the frequency of migraine occurrence on either Sunday or Monday of the time change weekend compared to Sunday or Monday before or Sunday or Monday after the time change. **Results**: Data from 258 patients were analyzed (86.8% women; average age: 51.5 years; average headache frequency: 7.7 days/month; 83.3% episodic migraine). Our results showed a significant increase of 6.4% in migraine frequency on the Sunday and/or Monday in the week after the time change in spring compared to the week before the change. In autumn, conversely, there was a significant reduction of 5.5% in migraine frequency on the Sunday and/or Monday one week after the time change compared to the week before the change. The factor responsible for the significant changes was the increase in migraines on Monday one week after the time change in spring and the decrease in migraines on Sunday one week after the time change in autumn. **Conclusions**: When switching from standard time to daylight savings time in the spring, the frequency of migraines increases significantly one week after the time change. In autumn, in comparison, there is an inverse trend with a reduction in migraine frequency. These data suggest that synchronization is disturbed when switching to daylight savings time. Conversely, synchronization normalizes in autumn. In view of the high prevalence of migraines, this can have extensive individual and social consequences.

## 1. Background

Migraine is characterized by episodic headache attacks lasting between 4 and 72 h. According to the International Classification of Headache Disorders ICHD-3, typical headache characteristics are unilateral localization, pulsating character, moderate-to-severe intensity, aggravation by routine physical activities, and the accompanying occurrence of nausea, vomiting, and/or hypersensitivity to light and sound [1]. If headaches occur at least 15 days a month and fulfill the criteria of migraine at least 8 days a month or respond to a specific migraine medication such as a triptan, the subform of chronic migraine is present [1]. In a representative study from 2020, the one-year prevalence of migraine in Germany was recorded as 14.8% in women and 6.0% in men [2]. In 2019, around 581,761,847 migraine cases were recorded worldwide, an increase of 16% compared to 1990 [3]. The pathophysiology of migraine is associated with activation of the trigeminovascular system [4], with both genetic and environmental influences being relevant risk factors [5,6]. Migraine is one of the ten most disabling disorders of all nervous system disorders [7]. In adults aged 20 to 59 years of age, migraine is the second most common cause of Disability-Adjusted Life Years (DALYs) lost due to illness or disability [7].

In a meta-analysis of trigger factors for migraine attacks, including 86 articles published from 1958 to 2015, 420 individual triggers were identified [8]. Stress (58%) and sleep (41%) comprised the most frequently mentioned triggers. Patients cited altered sleep patterns, a disturbed sleep rhythm, lack of sleep, oversleeping, sleep disturbances, naps, and weekend sleep as important triggers [8]. A regular sleep rhythm is therefore recommended as a preventive measure for migraine, with the aim of achieving a stable circadian rhythm and adequate sleep hygiene [9,10]. A possible effect of switching to daylight savings time is not known.

Around a quarter of the world’s population experiences a time change by one hour twice a year (daylight savings time, DST) due to local legal regulations. Social time is adjusted, with natural conditions such as dawn remaining unchanged [11]. In effect, this change represents a change in social clocks, whereas the physical time, e.g., dawn and dusk, remains unchanged. In Germany, the time has been changed twice a year since 1980. On the last Sunday in March, the clocks are set forward by one hour from Central European Time (CET) to daylight savings time. On the last Sunday in October, the clocks are then set back from daylight savings time to CET. The economic and energy policy objective of the time change was to save energy by adapting to the changing daylight hours throughout the year. However, biological and medical aspects are not taken into account by policymakers.

Although changes to the daily routine are known to influence migraine, the potential impact of biseasonal time changes has not been systematically studied. This study uniquely addresses this gap by analyzing the chronobiological effects of time change on migraine frequency. A better understanding of the relationship between time change and migraine could contribute to targeted prevention and treatment strategies. In fact, this externally imposed legal regulation interferes with biological sleep patterns and sleep rhythms and could therefore be relevant as a trigger for migraine attacks [8,9,10]. There are several possible mechanisms by which the time change could affect migraines. These include sleep disturbances, hormonal changes due to shifts in circadian rhythms, stress due to the need to adjust social time to physical time, and modification of the duration and type of daylight exposure. Daylight plays a role in the synchronization of biological clocks [11]. Studies indicate that the regulation of circadian rhythms does not adjust to daylight savings time and that seasonal adaptation is disrupted by the introduction of daylight savings time [11,12,13,14]. Such changes can result in a disruption of human seasonal biology; for example, the time change in the spring leads to a significant increase in the risk of heart attacks [15,16,17]. Research also shows that the number of fatal traffic accidents increases by 6% when the time changes in the spring [18] and medical errors by doctors increase by 18% in the first seven days after the time change [19]. The aim of this study was therefore to investigate whether the biseasonal time change has an influence on migraine.

## 2. Methods

### 2.1. Design

This study is a single-center, retrospective data collection. The Ethics Committee of the Faculty of Medicine at Kiel University approved this study (D402/23). The study was conducted in accordance with the 1964 Declaration of Helsinki and its subsequent revisions.

**Figure 1:**
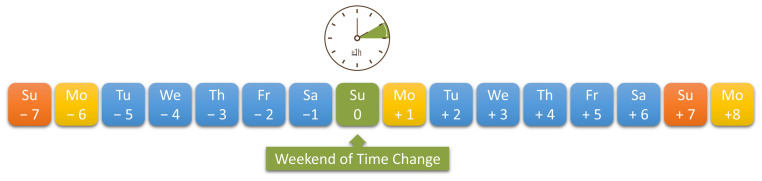
Recorded period from the Sunday before (−7 days) the time change (day 0) to the second Monday after the time change (day +8). Headache data were analyzed for Sunday (day 0) and Monday (day +1) of the actual time change weekend and for Sunday (day −7) and Monday (day −6) one week before and after the time change (day +7 and day +8).

The analysis included patients who were treated for episodic migraine with or without aura or chronic migraine in 2020, 2021, and 2022 at the Kiel Migraine and Headache Center, Germany, and who presented for regular follow-up in the first quarter of 2023. The diagnoses of episodic migraine with or without aura or chronic migraine had been confirmed in advance by qualified doctors at the center using ICHD-3 [1]. A prerequisite for inclusion in the analysis was the availability of systematically kept complete headache calendars for a time change period in the spring or autumn of 2020, 2021, or 2022. The headache calendars were kept independently of the study’s post hoc research question to prevent corresponding bias with the latter. The complete time change period (Figure 1) covered the period from the Sunday before (−7 days) the time change (day 0) to the second Monday after the time change (day +8). This factor meant that headache data were available for Sunday (day 0) and Monday (day +1) of the actual time change weekend and from Sunday (day −7) and Monday (day −6) one week before and after the time change (day +7 and day +8, respectively). A maximum of six time change periods could therefore be included in the evaluation for one patient if the headache calendars for all three years were available for the spring and autumn. The headache diaries were kept in either paper or digital form. The prerequisite was that the occurrence of migraine could be assigned to individual days.

An exclusion criterion was the presence of chronic migraine with daily occurrence of migraine attacks in the sense of *chronic daily headache*. Patients who could not reliably distinguish between a migraine and a tension-type headache were also excluded.

Data on the following variables were collected: age; gender; main migraine diagnosis (migraine without aura, migraine with aura, or chronic migraine); average migraine frequency (days/month) in the evaluated study periods of March/April and October/November in the years 2020, 2021, and 2022; the occurrence of migraine headaches on Sunday and Monday of the time change; the occurrence of migraine headaches on Sunday and Monday before the time change weekend; the occurrence of migraine headaches on Sunday and Monday after the time change weekend. The data extracted from the patient files were recorded pseudonymously.

### 2.2. Primary Outcome Measures

The primary outcome measure was the frequency of migraine occurrence on either Sunday (day 0) or Monday (day +1) of the time change weekend compared to Sunday (day −7) or Monday (day −6) before or Sunday (day +7) or Monday (day +8) after the time change. Average values for the years 2020, 2021, and 2022 were calculated separately for the three spring periods and the three autumn periods.

The three-year period was chosen to minimize possible climatic influences on migraine frequency. Based on the criteria of ICHD-3 [1], days with migraine and interval headaches are combined for the diagnosis of chronic migraine. By definition, headaches must occur at least 15 days per month. Of these, at least 8 days per month must fulfill the criteria of migraine with or without aura or respond to a specific migraine medication (triptan or ergotamine). In the present study, the influence of the time change on days with the characteristics of migraine, but not for interval headaches, was determined accordingly. In the case of chronic migraine, only migraine days and days on which triptans were effective were recorded.

In Germany, the time change always takes place on the last Sunday in March and the last Sunday in October of each year. In March, the clocks are set forward at 02:00 a.m. to 03:00 a.m.; in October, they are set back at 03:00 a.m. to 02:00 a.m. Trigger factors can trigger attacks up to two days later [20], which is why not only the Sunday but also the Monday after the time change was taken into account when defining the primary outcome measure. Accordingly, the comparison periods were also extended to the two days, Sunday and Monday.

### 2.3. Secondary Outcome Measures

- The average frequency of migraine occurrence on the Sunday of the time change weekend compared to the Sunday before and Sunday after the time change.
- The average frequency of migraine occurrence on the Monday of the time change weekend compared to the Monday before and the Monday one week after the time change.
- The frequency of migraine occurrence on either Sunday or Monday of the time change weekend compared to Sunday or Monday before or Sunday or Monday after the time change separately for patients with episodic migraine and those with chronic migraine.

For all secondary outcome measures, average values for the years 2020, 2021, and 2022 were calculated for the three spring periods and the three autumn periods.

### 2.4. Statistical Evaluation

The arithmetic mean and standard deviation were calculated for continuous variables. Categorical variables were recorded as absolute and relative frequencies. Fisher’s exact test was used to investigate differences in frequencies between groups. All the statistical tests were two-sided. A *p*-value of <0.05 was considered statistically significant. The analysis was carried out using Social Science Statistics Version 2023 software.

## 3. Results

### 3.1. Clinical Characteristics of the Study Population

**Figure 2:**
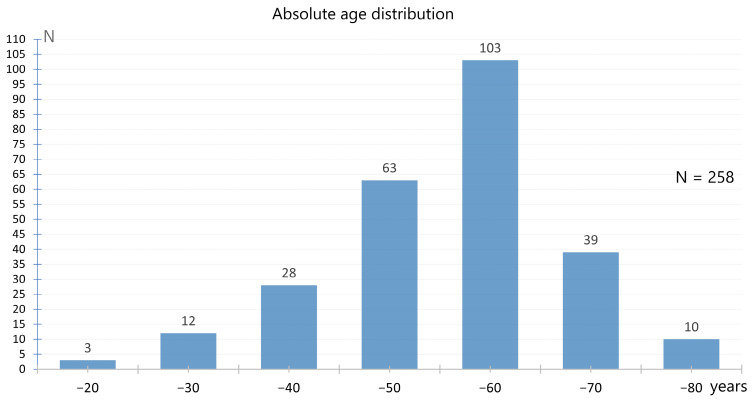
Absolute age distribution of the evaluated patients.

In total, data were collected from 258 patients, of whom 224 were women (86.8%) and 34 were men (13.2%). The average age was 51.54 ± 11.78 years (minimum 17 years; maximum 80 years). Most patients were in the fifth and sixth decade of life. The age distribution of the patients is shown in Figure 2.

The diagnostic criteria for episodic migraine according to ICHD-3 with or without aura were met by 215 patients (83.3%), and those for chronic migraine were met by 43 patients (16.7%).

The average frequency of migraine days per month (MMD, monthly migraine days) of all patients (*n* = 258) was 7.7 ± 3.5 days/month (minimum = 2; maximum = 20). In patients with episodic migraine, the number of monthly migraine days was 6.7 ± 2.4 days/month (minimum = 2; maximum = 14). For chronic migraine, the number of typical migraine days per month was 12.8± 3.8 days/month (minimum = 8; maximum = 20).

Medication-based migraine prophylaxis was used by 248 patients (96.1%). The majority of patients (N = 253; 98.1%) used triptans for the treatment of migraine attacks.

**Figure 3:**
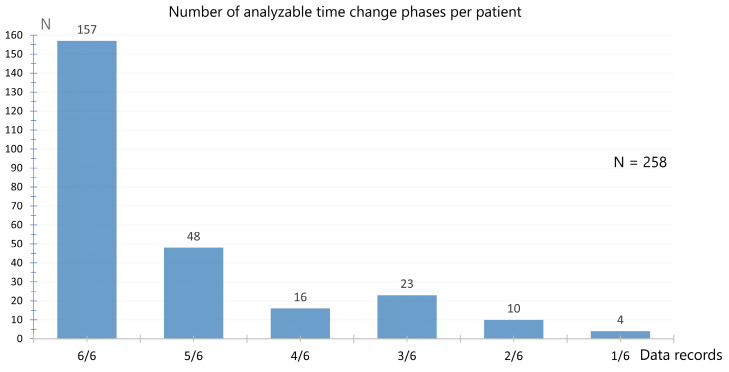
Number of evaluable time change phases per patient. Maximum of 6 phases recorded, spring and autumn 2020, 2021, and 2022. A complete data set of a time change consists of week −1, W0, and week +1.

Six complete data sets were derived from 157 patients (60.9%) from the time changes in the spring and autumn of 2020, 2021, and 2022. The frequencies of the evaluable time change phases recorded for the patients are shown in Figure 3.

### 3.2. Effect of the Time Change in Spring

**Figure 4:**
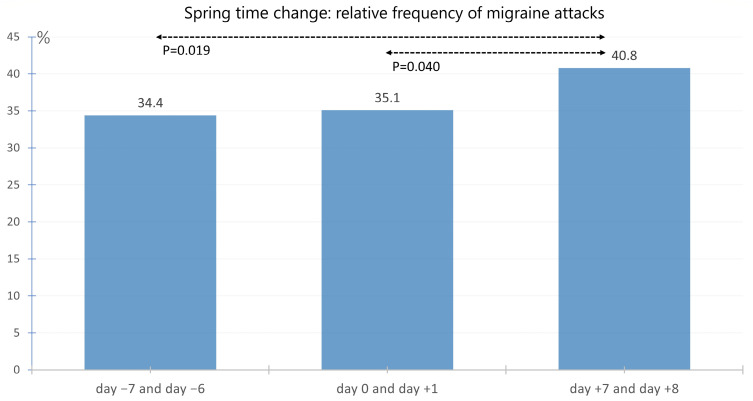
Average migraine frequency on Sunday and/or Monday at the 3 time points day −7 and day −6 (week before the time change), day 0 and day +1 (weekend of the time change), and day +7 and day +8 (week after the time change) in spring. The frequency of migraines on Sunday and/or Monday on day +7 and day +8 after the time change was significantly higher than on day −7 and day −6 before the time change (*p* = 0.019) and than on the time change weekend (day 0 and day +1) itself (*p* = 0.040).

The average frequency of migraines on the Sunday (day 0) of the spring time change or the Monday immediately following (day +1) in 2020, 2021, and 2022 was 35.1%. In the comparative period one week before the time change (day −7 and day −6), the average frequency was 34.4%, and in the week after the time change (day +7 and day +8), it was 40.8%. The frequency of migraines on Sunday and/or Monday on day +7 and day +8 after the time change was significantly higher than on day −7 and day −6 before the time change (*p* = 0.019) and also on the time change weekend (day 0 and day +1) itself (*p* = 0.040). The respective frequencies are shown in Figure 4.

### 3.3. Effect of the Time Change in Autumn

**Figure 5:**
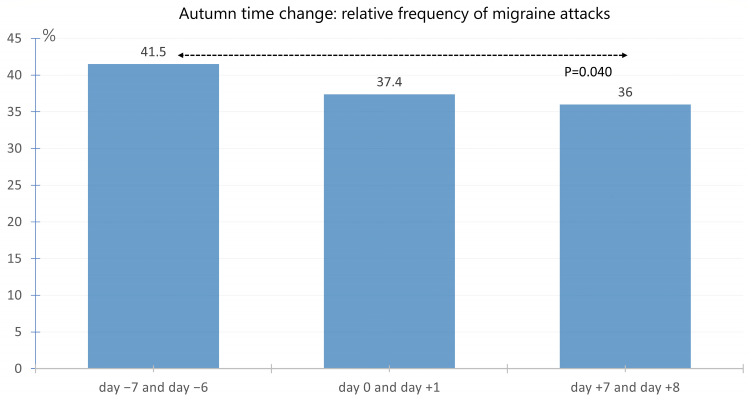
Average migraine frequency on Sunday and/or Monday at the time points day −7 and day −6 (week before the time change), day 0 and day +1 (weekend of the time change), and day +7 and day +8 (week after the time change) in autumn. The frequency of migraines on Sunday and/or Monday on day +7 and day +8 after the time change was significantly lower than on day −7 and day −6 before the time change (*p* = 0.040). The migraine frequency on the time change weekend day 0 and day +1 was lower than in the previous week on day −7 and day −6, but the difference was not significant (*p* = 0.14).

The average frequency of migraines on the Sunday or the Monday immediately following the time change in autumn in 2020, 2021, and 2022 was 37.4%. In the comparative period one week before the time change, the average frequency was 41.5%, and in the week after the time change, it was 36.0%. The frequency of migraines on Sunday and/or Monday on day +7 and day +8 after the time change was significantly lower than on day −7 and day −6 before the time change (*p* = 0.040). The frequency of migraines on day 0 and day +1 of the time change weekend was lower than on day −7 and day −6; however, the difference was not significant (*p* = 0.14). The respective frequencies are shown in Figure 5.

### 3.4. Migraine Frequency on the Sunday of the Time Change in Spring

The average frequency of migraines on the Sunday before, during, and after the time changes in spring was 21.6%, 23.3%, and 23.3%, respectively, with the frequencies not differing significantly.

### 3.5. Migraine Frequency on the Sunday of the Time Change in Autumn

The average migraine frequency on the Sunday before, during, and after the time change in autumn was 25.8%, 24.5%, and 21.0%, respectively. The migraine frequency on Sunday on day +8 after the time change was significantly lower than on day −7 before the time change (*p* = 0.041). Although the frequency of migraines on Sunday on day +8 after the time change was also lower than on day 0 before the time change, the difference was not significant (*p* = 0.139).

### 3.6. Migraine Frequency on the Monday of the Time Change in Spring

The average migraine frequency on the Monday before, during, and after the time change in spring was 21.5%, 21.9%, and 26.7%, respectively. The migraine frequency on Monday on day +8 after the time change was significantly higher than on day −6 before the time change (*p* = 0.03). Although the frequency of migraines on Monday on day +8 after the time change was also higher than on day 0, the difference was not significant (*p* = 0.053).

### 3.7. Migraine Frequency on the Monday of the Time Change in Autumn

The average frequency of migraines on the Monday before, during, and after the time changes in autumn was 25.5%, 23.6%, and 24.6%, respectively, with the frequencies not differing significantly.

### 3.8. Migraine Frequency During Time Change in Spring: Comparison of Chronic vs. Episodic

**Figure 6:**
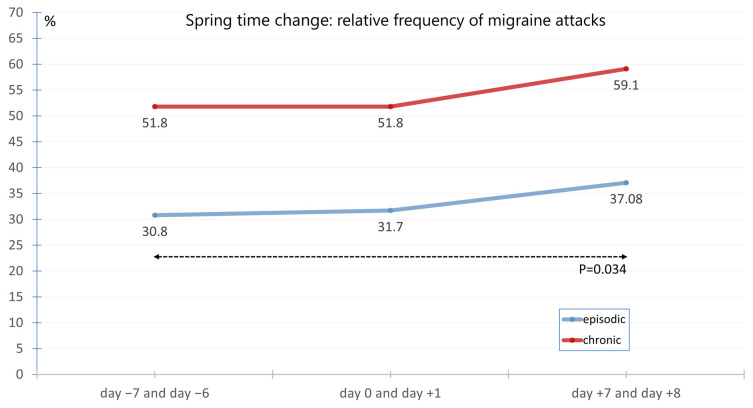
Average migraine frequency on Sunday and/or Monday at the 3 time points day −7 and day −6, day 0 and day +1 as well as time point day +7 and day +8 in spring in the presence of episodic migraine versus chronic migraine. There was a significant difference in episodic migraine between the time points day −7 and day −6 before the time change and day +7 and day +8 after the time change (*p* = 0.0342).

The average migraine frequency on Sunday and/or Monday before, during, and after the time changes in spring was 51.8%, 51.8%, and 59.1% for chronic migraine. The corresponding frequencies for episodic migraine were 30.8%, 31.7%, and 37.1% (Figure 6). The frequencies for chronic migraine did not differ significantly. In contrast, there was a significant difference in episodic migraine between the time points day −7 and day −6 before the time change and day +7 and day +8 after the time change (*p* = 0.0342). The difference between the time points day −7 and day −6 before the time change and the time points day +7 and day +8 after the time change shows a trend-like effect that is not statistically significant (*p* = 0.0734) but could indicate a possible association.

### 3.9. Migraine Frequency During Time Change in Autumn: Comparison of Chronic vs. Episodic

**Figure 7:**
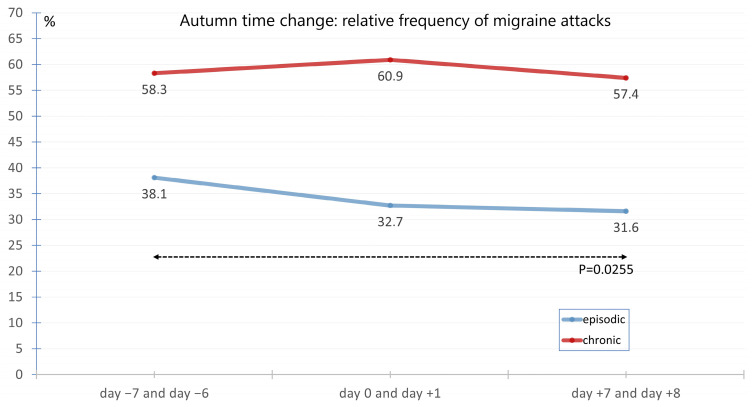
Average migraine frequency on Sunday and/or Monday at the 3 time points day −7 and day −6, day 0 and day +1 as well as time point day +7 and day +8 in autumn in the presence of episodic migraine versus chronic migraine. The frequencies for chronic migraine did not differ significantly. In contrast, there was a significant difference between the time points day −7 and day −6 before the time change and day +7 and day +8 after the time change for episodic migraine (*p* = 0.0255).

The average frequency of migraine on Sunday and/or Monday before, during, and after the time change in autumn was 58.3%, 60.9%, and 57%, respectively, for chronic migraine. The corresponding frequencies for episodic migraine were 38.1%, 32.7%, and 31.6% (Figure 7).

The frequencies for chronic migraine did not differ significantly. In contrast, a significant difference was found for episodic migraine between the time points day −7 and day −6 before the time change and day +7 and day +8 after the time change (*p* = 0.0255).

## 4. Discussion

In this study, the effect of the time changes in spring and autumn on the occurrence of migraine attacks was investigated. In spring, there was a significantly higher migraine frequency one week after the time change compared to the time change weekend and the weekend before the time change. If we examine the migraine frequency for Sundays and Mondays separately, we see that the change is essentially due to a significant increase in migraines on Mondays one week after the time change. In autumn, in comparison, an inverse course was found. Migraine frequency tended to decrease immediately on the weekend of the time change and significantly one week later compared to the weekend before the time change. Examining the weekdays separately, a significant difference was already evident on the Sunday of the weekend after the time change.

The results demonstrate that time change can significantly influence the occurrence of migraines. This effect does not occur immediately on the weekend of the time change but with a delay of one week. This finding indicates that the time change does not act as a direct trigger; rather, the slow synchronization between social time and circadian biological rhythm could cause this delay [11,12,14].

Our data show that the time change itself does not generally lead to an increase in migraine attacks. Only the time change in spring with a switch to daylight savings time is associated with a significant increase in migraine attacks. In contrast, the time change in autumn with the coupling of physical and biological time by returning to standard time led to a decrease in migraine frequency. This finding indicates that it is not the change in sleep rhythm that is relevant as a direct factor but rather that other factors must also be involved. A delayed synchronization of biological clocks, which occurs in opposite directions in spring and autumn, may be responsible for the connection. Accordingly, Roenneberg et al. [12] showed in a large study that the time of sleep on non-work days follows the seasonal course of dawn at standard time but not at daylight savings time. These data suggest that synchronization is disturbed when switching to daylight savings time. The above findings could provide an explanation for the increase in migraines that we observed during the time change in spring. Conversely, synchronization normalizes in autumn. This change could result in a delayed improvement in migraine frequency. Similarly, the sleep and activity timing of chronotyped subjects adapts easily to the end of daylight savings time in autumn [14]. This fact would also correspond to the reduction in migraine frequency that we observed during the autumn time change. However, the adaptation does not occur with the switch to daylight savings time in spring, especially in late chronotypes [14]. Disturbed synchronization could therefore be responsible for the delayed increase in migraine frequency. The findings support the assumption that the human circadian system does not adapt directly to daylight savings time; its seasonal synchronization to the changing photoperiods is disturbed by the introduction of daylight savings time and can have a negative effect on seasonal biology [11]. Given the ongoing discussions about abolishing daylight savings time in several countries, our findings provide new evidence supporting the potential health benefits of maintaining a stable time system.

The pathophysiological basis for the connection between the biseasonal time change on migraine has not yet been clarified. In recent years, studies on chronobiology and the influence of circadian rhythms have been published. Mammals have a constant circadian rhythm, the pacemaker of which is located in the suprachiasmatic nucleus, a part of the ventral hypothalamus [21]. In the squirrel monkey (Saimiri sciureus), a lesion of this pacemaker leads to the abolition of circadian rhythm with an extension of sleep phases [21]. The pacemaker is continuously synchronized with daylight. This process takes place via direct innervation by retinal ganglion cells. The constant sleep–wake rhythm has a significant influence on numerous bodily functions [22]. Migraine attacks begin with a prodromal phase [1]. One to two days before the onset of headache, patients describe cravings, tiredness, yawning, hypersensitivity to stimuli, and water retention [23]. As a correlate of this prodromal phase, hypothalamic activation has been shown to occur 48 h before the onset of the actual headache phase [24]. It is important to note that the activation of the hypothalamus precedes the migraine headache phase. The hypothalamus has a variety of functions in the organism. In addition to the regulation of sleep, these include the regulation of appetite, thirst, and the autonomic, endocrine, and cardiovascular systems [25]. The neuropeptides orexin A and B produced in the hypothalamus play a role both in circadian rhythm and in the pathophysiology of migraine [25]. Dysfunction in orexin metabolism has been shown in migraine patients [26,27,28,29,30,31,32,33]. Episodic migraine is associated with reduced orexin levels in the cerebrospinal fluid, whereas chronic migraine and headaches caused by overuse of medication are associated with increased orexin levels. The clinical picture of narcolepsy points to a direct link between migraine and circadian rhythm via orexin [34,35]. In narcolepsy, autoimmunologically induced destruction of orexin-producing neurons in the hypothalamus is noted. Compared to the general population, patients with narcolepsy are significantly more likely to also suffer from migraines: 64% of women with narcolepsy and 45% of men [36]. The orexin system in the hypothalamus could therefore be a possible pathophysiological explanation for the connection between the time change and the occurrence of migraines.

The present study has some limitations. Our study cohort is not a representative sample of the population as a whole. The data were collected from patients at a tertiary headache center; thus, the severity of the condition will therefore be higher than in the overall group of sufferers. However, the relative increase in headache frequency is lower in high-frequency or chronic migraine than in patients with low headache days per month. It is therefore possible that the effect is underestimated. The vast majority of those examined (96.1%) were taking migraine prophylaxis medication. The aim of prophylaxis is to make the patient less sensitive to migraine triggers. For this reason, it is therefore possible that the influence of the time shift is underestimated in the patients studied. The effects may be higher in a population with only low rates of prophylactic use. For this reason, further studies on patients with episodic migraine in a representative population without preventive migraine medication would be of interest.

## 5. Conclusions

When switching from standard time to daylight savings time in spring, there was a significantly higher frequency of migraines in our study population one week after the time change. In contrast, the time change in autumn was associated with a decrease in migraine frequency. The results support the assumption that the human circadian system adapts incompletely to daylight savings time. The seasonal synchronization with the changing photoperiods could be disturbed by the introduction of daylight savings time, which could have a negative impact on seasonal biology. In view of the high prevalence of migraines, this could have extensive individual and social consequences. These findings underscore the need for further investigation into the long-term effects of time changes on neurological health. Future studies in representative patient populations without migraine prophylaxis could further elucidate the chronobiological effects of the biseasonal time change on migraine attacks.

## Data Availability

The original data presented in the study are openly available in Zenodo at https://doi.org/10.5281/zenodo.14792905.
